# *Erwinia plantamica* sp. nov., a Non-Phytopathogenic Bacterium Isolated from the Seedlings of Spring Wheat (*Triticum aestivum* L.)

**DOI:** 10.3390/microorganisms13030474

**Published:** 2025-02-20

**Authors:** Anna Egorshina, Mikhail Lukyantsev, Sergey Golubev, Eugenia Boulygina, Irina Khilyas, Anna Muratova

**Affiliations:** 1 Research and Development, Organic Park LLC, 420095 Kazan, Russia; egorshina.a.a@bionovatic.ru (A.E.); lukyantsev.m.a@bionovatic.ru (M.L.); 2 Institute of Biochemistry and Physiology of Plants and Microorganisms, Saratov Scientific Centre of the Russian Academy of Sciences (IBPPM RAS), 410049 Saratov, Russia; sngolubev@rambler.ru; 3 Institute of Fundamental Medicine and Biology, Kazan (Volga Region) Federal University, 420021 Kazan, Russia; boulygina@gmail.com (E.B.); irina.khilyas@gmail.com (I.K.)

**Keywords:** *Erwinia plantamica*, *Triticum aestivum*, *Hordeum vulgare*, plant-friendly

## Abstract

*Erwinia* are widely known as phytopathogenic bacteria, but among them, there are also plant-friendly strains that can promote plant growth (PGPR). The *Erwinia*-like strain OPT-41 was isolated from *Triticum aestivum* seedlings as a potential PGPR. The cells (0.9–1.3 × 1.5–3.1 µm) of this microorganism are Gram-negative, rod-shaped, motile (with peritrichous flagella), and non-spore- and non-capsule-forming. The 16S rRNA gene sequence analyses showed it is located in the *Erwiniaceae* family and has a pairwise similarity above the species delineation threshold of 98.65% with several of its members: *Erwinia tasmaniensis* (99.21%), *Candidatus* Pantoea bathycoeliae (98.93%), *Pantoea agglomerans* (98.87%), *Erwinia endophytica* (98.83%), *Erwinia persicina* (98.82%), *Erwinia billingiae* (98.76%) and *Erwinia aphidicola* (98.75%). Whole genome-based taxonomy performed on the Type (Strain) Genome Server clarified the status of strain OPT-41, detecting it as a potential new species in the genus *Erwinia*. The microorganism under study was the most closely related to the type strain of *E. phyllosphaerae*, demonstrating 27.2% similarity in dDDH, 83.44% similarity in OrthoANIu, and 1.9% difference in G+C content. The major fatty acids of strain OPT-41 were 9 C_16:1_, C_14:0_, and C_16:0_. A combination of genome-based taxonomy and traditional polyphasic taxonomy clearly indicated that strain OPT-41 belongs to a novel *Erwinia* species, for which the name *E. plantamica* sp. nov was proposed. OPT-41 (=IBPPM 712=VKM B-3873D=CCTCC AB 2024361) has been designated as the type strain. In addition, OPT-41 was found to have low degradation potential for host plant pectins and proteins and be friendly in *Triticum aestivum* and *Hordeum vulgare* crops.

## 1. Introduction

The genus *Erwinia*, first described by Winslow et al. with *Erwinia amylovora* as the type species [1], belongs to the family *Erwiniaceae* of the order *Enterobacterales* within the class *Gammaproteobacteria* [2], which is part of the phylum *Pseudomonadota* [3]. According to the List of Prokaryotic Names with Standing in Nomenclature (LPSN) resource [4] hosted at https://lpsn.dsmz.de (accessed on 1 November 2024), the number of species of this taxon with a valid and correct name is 18, and the total number of species is 47.

*Erwinia* are widely known as bacteria that cause plant diseases. First of all, they are the best-studied species, including *E. amylovora* [1] and *E. pyrifoliae* [5], the fire blight pathogens in the *Rosaceae* trees. As for the others, *E. mallotivora* [6] infects *Mallotus japonicus* (but not Japanese pear, peach, apple, plum, apricot, and chestnut trees), producing small, dark-brown spots on leaves as well as shoot blight. *E. psidii* [7] is the causative agent of the disease known as “guava bacterial blight” that limits *Psidium guajava* L. production in Brazil [8]. *E. billingiae* members act as secondary invaders [9], but Vidaurre-Barahona et al. [10] reported that these microorganisms can cause the bacterial canker of mango (*Mangifera indica*). *E. papaya* is the bacterial stem canker pathogen of *Carica papaya* [11]. *E. piriflorinigrans* [12] causes the blossom necrosis of pear (but not of other *Rosaceae*) and elicits a hypersensitive reaction when inoculated in tobacco and tomato leaves. *E. uzenensis* [13] is a bacterial black shoot pathogen of *Pyrus communis* L. Some strains of *E. persicina*, the species described by Hao et al. [14], are known to cause soft rot on carrots, garlic, and white and yellow onions [15]. The recently described species *E. pyri* [16] is responsible for the occurrence of a bacterial dieback disease in pear trees, a condition with similarities to pear fire blight.

There are also non-pathogenic *Erwinia* species. In particular, *E. tasmaniensis* [17] does not act as a pathogen with regards to *Rosaceae* trees. According to [18], *E. endophytica* strains also failed to show any activity as potato pathogens under the conditions tested. For some species, such as *E. oleae* [19] and *E. phyllosphaerae* [20], no evidence of phytopathogenicity has been reported. Importantly, among the members of such non-phytopathogenic or conditionally non-phytopathogenic species, strains with plant growth-promoting traits have been found. In particular, *E. endophytica* BSTT30^T^ and BSTT40 produce inorganic phosphate solubilizers and siderophores [18] and *E. phyllosphaerae* CMYE1^T^, inorganic phosphate solubilizers and indole-3-acetic acid [20]. Moreover, successful experiments on the influence of *Erwinia* inocula on the enhancement of the growth and yield of tomato [21], wheat [22], and ginseng [23] are shown. These facts indicate the prospects of using non-phytopathogenic *Erwinia* strains to stimulate the growth of food and medicinal plants.

In addition, some *Erwinia* species have been shown to be associated with insects: *E. aphidicola* with the pea aphid (*Acyrthosiphon pisum*) [24]; *E. typographi* with the bark beetle (*Ips typographus*) [25]; *E. teleogrylli* with the Chinese cricket (*Teleogryllus occipitalis*) [26]; and *Candidatus* E. impunctatus with the Highland midge (*Culicoides impunctatus*) [27]. The endo- and ectophytic species in the genus under discussion are not restricted to their insect hosts, having plant hosts as well [27]. Documented evidence of interactions of *Erwinia* bacteria with fungi [28], animals [29] or humans [30,31,32] is less common in the modern scientific literature.

During the search for bacteria with the ability to promote the growth of wheat and other cereal crops, the *Erwinia*-like strain OPT-41 isolated from *Triticum aestivum* seedlings was selected as promising. We assumed that the isolated strain would indeed be a *Erwinia* member. Moreover, it may well be friendly to cereal crops since such precedents have already been recorded. In this regard, the objectives of this study were to identify strain OPT-41 at the species level and test it for pathogenicity against wheat and barley.

## 2. Materials and Methods

### 2.1. Microorganism

This study was conducted with the *Erwinia*-like OPT-41 strain, which was isolated as a plant growth-promoting bacterium from spring wheat (*Triticum aestivum* L., var. Simbircite) seedlings in Kazan (Russia).

The OPT-41 strain was deposited in the Collection of Rhizosphere Microorganisms of the IBPPM RAS (WDCM no. 1021, Saratov, Russia, http://collection.ibppm.ru, accessed on 17 February 2025), the All-Russian Collection of Microorganisms (WDCM no. 342, Pushchino, Moscow Region, Russia, https://www.vkm.ru, accessed on 17 February 2025), and the China Center for Type Culture Collection (WDCM no. 611, Whuhan, Hubei, P.R.China, http://www.cctcc.org, accessed on 17 February 2025) as IBPPM 712, VKM B-3873D, and CCTCC AB 2024361, respectively.

### 2.2. Species Identification

The identification of the OPT-41 strain at the species level was carried out using whole genome-based taxonomy in combination with standard polyphasic taxonomy [33].

#### 2.2.1. 16S rRNA Gene Sequence Analysis

The 16S rRNA gene sequence analysis was performed on the GGDC web server (http://ggdc.dsmz.de, accessed on 15 July 2024; [34]), using the DSMZ phylogenomics pipeline [35] adapted to single genes as described in [36].

#### 2.2.2. Whole Genome-Based Taxonomic Analysis

Whole genome-based taxonomic analysis was performed on the Type (Strain) Genome Server (TYGS), a free bioinformatics platform hosted at https://tygs.dsmz.de (accessed on 15 July 2024) and operating in tandem with the List of Prokaryotic Names in Nomenclature (LPSN) database [34,37]. The details of this analysis, which included steps such as (1) the determination of closely related type strains, (2) the pairwise comparison of genome sequences, (3) phylogenetic inference, and (4) type-based species and subspecies clustering, are described in [36]. The 16S rRNA gene sequence required to determinate closely related type strains was extracted from the target genome using RNAmmer 1.2 [38]. Average nucleotide identity (ANI) calculation was carried out at https://www.ezbiocloud.net/tools/ani (accessed on 18 July 2024) using the OrthoANIu algorithm [39].

#### 2.2.3. Cultural, Morphological, Physiological, and Biochemical Traits

Growth tests were performed at 29 °C for 7 days on different media, including R2A agar, nutrient agar (NA), tryptic soy agar (TSA), and meat peptone broth (MPB). The growth temperature range was tested in MPB at 4, 5, 10, 29, 37, and 44 °C for 7 days. Tolerance to NaCl was detected in MPB supplemented with 0, 1, 2.5, and 6.5% (*w*/*v*) NaCl concentrations at 29 °C for 7 days. The pH range of growth was examined in buffered R2A broth from pH 4.0 to 10.0. Gram stain reaction was performed using the KOH lysis method [40].

Cell morphology was examined by light and transmission electron microscopy after 2 days of incubation on R2A agar at 29 °C. The microscopy of bacterial cells was executed using a Leica DM2500 light microscope (Leica Microsystems GmbH, Wetzlar, Germany). Transmission electron microscopy (TEM) images were recorded on a Libra-120 transmission electron microscope (Carl Zeiss, Oberkochen, Germany) with an accelerating voltage of 120 kV at the Simbioz Center for the Collective Use of Research Equipment in the Field of Physico-Chemical Biology and Nanobiotechnology, IBPPM RAS, Saratov. Copper grids coated with a formvar film were used as substrates. In total, 5 µL of an aqueous suspension of bacteria was applied to the substrate and kept for 30 min. Bacteria were characterized by size using the iTEM application.

Biochemical tests were conducted according to [41,42]. The API^®^20NE kits (bioMerieux, Marcy l’Etoile, France) were also used according to the manufacturer’s instructions.

#### 2.2.4. Chemotaxonomic Analysis

For chemotaxonomic characterization, strain OPT-41 was cultivated on R2A plates at 28 °C for 2 days. Cellular fatty acids were extracted and analyzed by using gas chromatography (GC) according to the protocol of the Sherlock Microbial Identification System [43]. A GC-2010 (Shimadzu Corporation Analytical Instruments Devision, Kyoto, Japan) chromatograph equipped with an SPB^®^-5 (30 m × 0.32 mm × 0.25 µm) (Supelco, Bellefonte, PA, USA) capillary column applying the temperature program of 130 °C to 270 °C was used. The evaporator temperature was 270 °C, AND the flame ionization detector (FID) temperature was 300 °C. Fatty acids were identified using the standard mixture of the fatty acid methyl esters (Supelco 37 Component FAME Mix, Supelco, Bellefonte, PA, USA).

### 2.3. DNA Extraction and Sequencing

#### 2.3.1. Genomic DNA Extraction

Genomic DNA extraction from bacterial cells was performed using the commercial ZymoBIOMICS DNA Miniprep Kit (Zymo Research Europe GmbH, Freiburg im Breisgau, Germany) according to the manufacturer’s protocol with minor modifications. The overnight cell culture grown on the LB medium was centrifuged in a high-speed centrifuge at 13,000 rpm in a volume of 1 mL for 1–2 min. The supernatant was decanted, and the pellet was suspended in 160 μL of 10 mM Tris-HCl (pH 8.0), and then 40 μL of lysozyme with a concentration of 20 mg/mL was added. The resulting mixture was blended and incubated in a thermostat for 1 h at 37 °C. Then, 750 μL of ZymoBIOMICS Lysis Solution was added to the mixture, and afterwards, DNA was isolated as described in the manufacturer’s protocol. DNA concentration was determined using a Qubit 2.0 fluorimeter (Invitrogen Life Technologies Holdings Pte Ltd., Singapore). Its integrity was assessed using electrophoresis in 0.8% agarose gel and staining with 1% ethidium bromide solution (neoFroxx GmbH, Einhausen, Germany).

#### 2.3.2. Shotgun Library Construction

To prepare the whole-genome shotgun library, genomic DNA was firstly sonicated using a Covaris S220 device, and then it was treated with a NEBNext Ultra II kit (NEB, Ipswich, MA, USA) according to the manufacturer’s instructions. The quantitative and qualitative assessment of the resulting library was performed using a Qubit 2.0 fluorimeter (Invitrogen) and on High Sensitivity DNA Kit chips of the 2100 Bioanalyzer (Agilent, Waldbronn, Germany).

#### 2.3.3. Whole-Genome Sequencing and Assembly

Whole-genome sequencing was carried out on an Illumina MiSeq platform in paired-end reading mode (2 × 300) with the MiSeq Reagent Kit v3 (Illumina, San Diego, CA, USA). The removal of adapter sequences from reads was performed using FASTP v. 0.23.2 software [44]; and de novo genome assembly was carried out using SPAdes v. 3.15.3 [45]. The OPT-41 whole-genome shotgun project was deposited at GenBank under the accession JBGCUC000000000 (BioProject accession, PRJNA1137566; BioSample accession, SAMN42622146; assembly accession, GCA_043420595.1).

#### 2.3.4. 16S rRNA Gene Amplification and Sequencing

The 16S rRNA gene sequence was determined using conventional PCR amplification and Sanger sequencing. The same pair of primers, 16S-8-f-B 5′-AGRGTTTGATCCTGGCTCA-3′ and 16S-1350-r-B 5′-GACGGGCGGTGTGTACAAG-3′ [46], was applied for both amplification and sequencing. Sequencing was performed on an ABI Prism 3500 Genetic Analyser (Applied Biosystems Inc., Waltham, MA, USA) in accordance with the manufacturer’s protocol. The sequence was deposited at GenBank under the accession PQ039748.

### 2.4. Extracellular Enzymatic Activity Analysis

The activities of extracellular pectate lyase, polygalacturonase, protease, and cellulase were measured in culture supernatants of the studied strain OPT-41 and the virulent strain of *Pectobacterium atroseptcium* SCRI1043 (ATCC BAA-672) (formerly *Erwinia carotovora* ssp. atroseptica SCRI1043) after one and two days of cultivation. The strains were cultured at 28 °C with aeration (180 rpm) in a minimal medium (MM) containing 50 mM of potassium phosphate buffer (pH of 7.0), 7.6 mM of (NH_4_)_2_SO_4_, 1.7 mM of MgCl_2_, 1.7 mM of NaCl, and 2.0 g/L of pectin (FLUKA Biochemika, Buchs, Switzerland), supplemented with 1/1000 *v*/*v* wheat leaf extract. The wheat leaf extract was prepared as follows: One hundred grams of fresh leaves of wheat plants, grown in vermiculite under a 16/8 light/dark cycle for 14 days, were ground in three volumes (*w*/*v*) of distilled water. The resulting suspension was filtered through gauze, and the remaining debris was ground in two volumes of water and filtered again. The two portions of the obtained filtrates were combined and centrifuged at 10,000 g for 10 min at room temperature. The supernatant was collected, incubated for 10 min at 80 °C and centrifuged again. The extract was then sterilized through nitrocellulose filters with 0.22 μm pores (Corning Inc., Corning, NY, USA) under sterile conditions and stored frozen at −20 °C until use.

The enzymatic activities were measured as previously described [47,48]. Briefly, the cellulase (endoglucanase) and polygalacturonase activities were determined by measuring the reducing sugars released after the enzymatic hydrolysis of the corresponding substrates: carboxymethyl cellulose (Sigma-Aldrich, Burlington, MA, USA) and polygalacturonic acid (Sigma-Aldrich, USA), respectively. The DNS reagent (Sigma-Aldrich, USA) was used to measure the reducing sugars at 540 nm. The amount of enzyme releasing 1 μmol of reducing sugars min^−1^ per 10^8^ bacterial cells was defined as one unit (U) of cellulase or polygalacturonase activities. Protease activity was assessed at 440 nm using azocasein (Sigma-Aldrich, USA) as a substrate. One unit (U) of protease activity was defined as the amount of enzyme required to produce an absorbance change of 1.0 per min per 10^8^ bacterial cells. Pectate lyase activity was determined by measuring the degradation of polygalacturonic acid (Sigma-Aldrich, USA) into unsaturated products at 234 nm. The amount of enzyme releasing 1 μmol of unsaturated products min^−1^ per 10^8^ bacterial cells was defined as one unit of pectate lyase activity. The absorbance in all enzymatic assays was measured using a CLARIOstar microplate reader (BMG Labtech GmbH, Ortenberg, Germany). Activities were assayed in three (cellulase and polygalacturonase), four (protease), and seven (pectate lyase) biological replicates. All measurements were performed at least in triplicate.

### 2.5. Effect of Bacterial Inoculation on Seed Germination

In order to evaluate the effects of the OPT-41 strain, the seeds of soft wheat (*Triticum aestivum* L., var. Ekada 282) and spring barley (*Hordeum vulgare* L., var. Vakula) were used. Preliminary seeds were vernalized at +4 °C for 18 h. Then, vernalized seeds were surface-sterilized in 70% ethanol for 1 min and 50% bleach with 0.001% Triton X-100 for 30 min and rinsed several times with sterile tap water. The OPT-41 strain was then inoculated in Luria–Bertani broth (LB) at 200 rpm for 18 h. The culture was then subjected to centrifugation and rinsed with sterile tap water twice. The bacterial suspension was prepared at a concentration of 1.1 × 10^7^ CFU/mL, after which the seeds were incubated at 30 °C and 200 rpm for 3 h. The incubation of the seeds in sterile tap water served as the control condition. In total, 400 seeds from each treatment were then placed on sterile filtered paper soaked with 5 mL of water in Petri dishes and stored at room temperature in the dark for 5 days. The seeds were then subjected to an incubation period in a growth chamber under a 16 h light/8 h dark cycle at 20 °C for 2 days. The germinated seeds were counted on the 3rd and 5th days. The incubation was maintained for a period of 7 days, during which the following parameters were recorded: germination energy (3 days), final germination (5 days), shoot and root length, fresh and dry weight, and fungal disease incidence (7 days).

### 2.6. Statistics

A statistical analysis of the data regarding the extracellular enzymatic activities of bacteria, as well as the morphological and physiological parameters of plants, was performed using GraphPad Prism v. 8.0.1 (GraphPad Software, LLC, San Diego, CA, USA). The significance of differences between the two groups of data was assessed using the unpaired *t* test and two-tailed Mann–Whitney test (*p* < 0.05).

## 3. Results and Discussion

### 3.1. Species Identification of Strain OPT-41

#### 3.1.1. 16S rRNA Gene-Based Taxonomy

The 16S rRNA gene sequence of strain OPT-41 was received in two ways: (i) PCR amplification followed by Sanger sequencing (PQ039748) and (ii) extraction from the whole genome (JBGCUC000000000). Their lengths are 1342 and 1542 bp, respectively, and they have 100% similarity in the overlap region.

**Figure 1:**
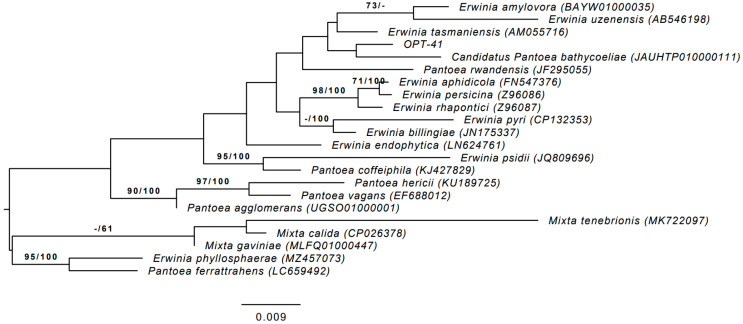
Midpoint-rooted maximum-likelihood tree based on the 16S rRNA gene sequences from strain OPT-41 and its phylogenetic neighbors. The evolutionary model used is GTR+GAMMA. Maximum-likelihood (**left**) and maximum parsimony (**right**) bootstrap values (>60%) are shown near the branches. GenBank accession numbers are in parentheses. Bar, 0.009 substitutions per nucleotide position.

The phylogenetic relationships between strain OPT-41 and its relatives are shown in Figure 1. As can be seen, the strain of interest is located in the *Erwiniaceae* family, forming a clade with *Candidatus* Pantoea bathycoeliae, *Erwinia tasmaniensis*, *Erwinia amylovora*, and *Erwinia uzenensis*. At the same time, strain OPT-41 has the highest 16S rRNA gene sequence similarity with *Erwinia tasmaniensis* (99.21%), *Candidatus* Pantoea bathycoeliae (98.93%), *Pantoea agglomerans* (98.87%), *Erwinia endophytica* (98.83%), *Erwinia persicina* (98.82%), *Erwinia billingiae* (98.76%) and *Erwinia aphidicola* (98.75%). With the remaining relatives, this similarity is below the 98.65% threshold suggested for species differentiation [49].

#### 3.1.2. Whole Genome-Based Taxonomy

**Figure 2:**
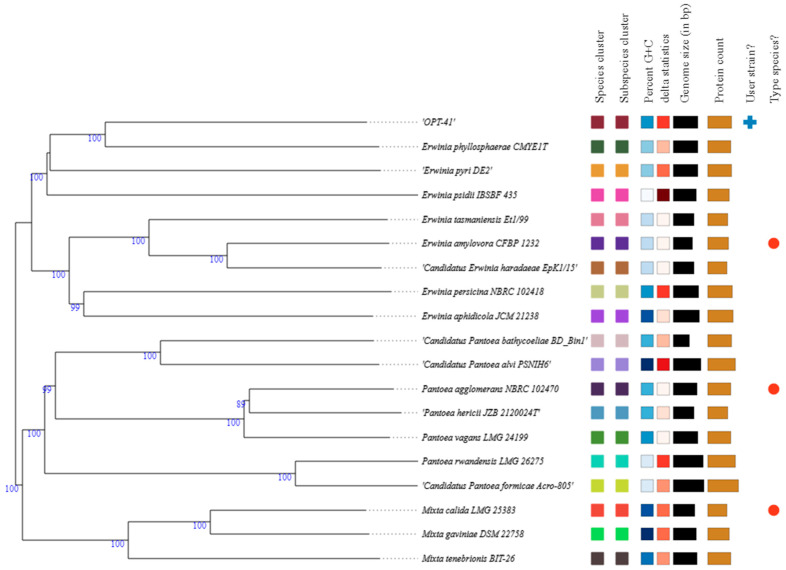
Midpoint-rooted minimum evolution tree based on the whole-genome sequences from strain OPT-41 and its phylogenetic neighbors. The intergenomic distances are calculated by using the GBDP approach. Pseudo-bootstrap values (>60%) based on 100 replications are shown at branch nodes (average branch support, 96.4%).

The TYGS type strains closely related to strain OPT-41 covered 18 members of the *Erwiniaceae* family, 8 of which belong to the genus *Erwinia*; 7 to the genus *Pantoea*; and 3 to the genus *Mixta*. Details of these strains are in Table S1. A phylogenetic tree of strain OPT-41 and its close relatives, as well as other genome-derived information, is showed in Figure 2.

According to this figure, clustering yielded 19 species and 19 subspecies clusters. At the same time, strain OPT-41 did not share either a species or subspecies cluster with other microorganisms. In the phylogram, the strains under consideration form three clearly separated genus clades, with strain OPT-41 included in the *Erwinia* clade, demonstrating the closest relationship with the type strain of *E. phyllosphaerae*. Notably, this relationship between OPT-41 and the *E. phyllosphaerae* type strain is not supported in the 16S rRNA gene-based phylogenetic tree. Within the order *Enterobacterales*, similar observations have also been noted by other authors [2], including when *E. phyllosphaerae* itself was described as a new species [20]. According to [2], (i) phylogenetic trees based on the 16S rRNA gene sequence have a limited ability to resolve clades identified in the genome-based phylogenetic trees; and (ii) the branching of the *Enterobacterales* genera and species in 16S rRNA gene-based phylogenies shows significant stochasticity depending on the algorithms used and the bacteria analyzed. Pairwise comparisons of the OPT-41 genome against closely related type strain genomes in terms of dDDH, OrthoANIu, and difference in G+C content are summarized in Table 1. As follows from it, the target strain has the best similarity to the *E. phyllosphaerae* type strain. In particular, the dDDH value computed using formula *d_4_* recommended for incomplete draft genomes [34,50] is 27.2% and the OrthoANIu value is 83.44%. Both the first and second values are below the species threshold of 70% [37] and 95–96% [51], respectively. Both dDDH and ANI are among the most widely used overall genome relatedness indices (OGRIs) for in silico species delineation; however, the dDDH approach outperforms the ANI approach [34]. The G+C content difference between the OPT-41 genome and the *E. phyllosphaerae* type strain genome is 1.9%. Within species, G+C content varies no more than 1% if computed from genome sequences [52]. These facts indicate that the OPT-41 strain most likely represents a novel species within the genus *Erwinia*.

#### 3.1.3. Cultural, Morphological, Physiological, and Biochemical Characteristics

**Figure 3:**
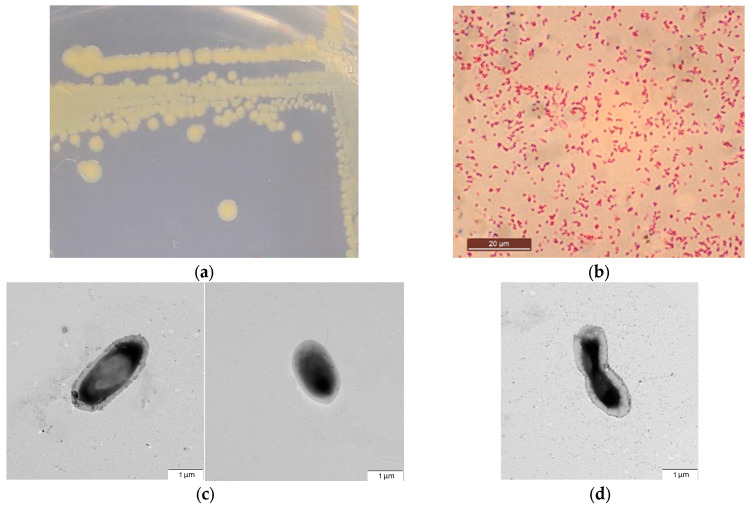
Colonies and cell morphology of strain OPT-41: (**a**) 24 h colonies on TSA medium; (**b**) Gram-negative rods in 24 h culture; (**c**) transmission electron microscopy of single cell grown on R2A for 24 h; (**d**) transmission electron microscopy of cell division; peritrichous flagella is also seen.

After 1–2 days of incubation at 29 °C, colonies of OPT-41 on R2A agar were circular, smooth, convex, yellowish, translucent and about 3–5 mm in diameter, they did not produce a pigment diffusing into agar. (Figure 3a). When grown in liquid medium, they have uniform turbidity on the medium. They can grow in the presence of 2.5 NaCl but not in the presence of 6.5 NaCl. The pH range for growth was 4.5–8.5 and the optimum pH was 6.5–7.0. The range temperature for growth was 5–44 °C and the optimum temperature was 29 °C.

In 24 h culture grown on R2A agar, rod-shaped single Gram-negative cells (0.9–1.3 × 1.5–3.1 µm) are present (Figure 3b–d). They are mobile and do not form spores or capsules.

According to the testing, the OPT-41 strain was catalase-positive and oxidase- and urease-negative. It produced nitrate reductase and acetoin did not produce arginine hydrolase, lysine decarboxylase, lipase, lecithinase, and indole. It hydrolyzed esculin, gelatin and starch. In the OF test, acid produced from the fermentation was observed with glucose, rhamnose, mannose, lactose, maltose, sucrose, cellobiose, arabinose, melibiose, inositol, and sorbitol. Gas formation was observed with maltose, mannose, melibiose, lactose, inositol, and sorbitol.

According to API^®^ 20NE tests, this strain produced β-galactosidase, assimilated D-glucose, L-arabinose, D-mannose, D-mannitol, D-maltose, N-acetylglucosamine, potassium gluconate, malic acid, and sodium citrate but did not assimilate capric, adipic acid and phenylacetic acids (Figure S1).

#### 3.1.4. Chemotaxonomy

The major fatty acids of strain OPT-41 (>10% of the total amounts) were 9 C_16:1_ (21.1%), C_14:0_ (23.4%), and C_16:0_ (53.5%) (Table 2). The fatty acid profile of this strain differed from that of the closest type strain *E. phyllospherae* CMYE1 [20] mainly by the presence of 9 C_16:1_ and the absence of C_17:0_ cyclo, C_14:0_ 3-OH and/or C_16:1_ iso I (Summed feature 2), and C_16:1_ ω7c and/or C_16:1_ ω6c (Summed feature 3).

#### 3.1.5. Differential Characteristics

Characteristics that distinguish strain OPT-41 from the closely related type strains of *Erwinia* species are summarized in Table 3. All these characteristics are common among *Erwinia*, although not all strains of *Erwinia* species possess them or information about the presence/absence of a particular characteristic is not yet available. Compared to *E. phyllospherae* CMYE1^T^, the most closely related type strain, the bacterium under study, has a larger cell size, does not grow at 4 °C, does not produce indole, does not hydrolyze arginine, but hydrolyze gelatin and starch, and can produce acid from the fermentation of maltose, D-lactose, cellobiose, and sorbitol. In addition, it has a higher G+C content. Comparisons of the OPT-41 strain with other closely related type strains show other sets of distinctive features. Nevertheless, there are three characteristics by which the studied strain can be clearly distinguished from closely related type strains. These are cell size, gelatin hydrolysis and the genomic DNA G+C content. It is important to note that the relatively large cell size of the OPT-41 strain is not a unique feature, since among the *Erwinia* species, there are members with a similar cell size: *E. typographi* Y1^T^ (0.7–0.8 × 2.0–4.0 µm) [25] and *E. oleae* DAPP-PG 531^T^ (0.9 × 1.5–3.0 µm) [19]. In addition to the strain under study, the ability to hydrolyze gelatin was also found in *E. amylovora* CFBP 1232^T^ [25], *E. uzenensis* YPPS 951^T^ [13], and *E. oleae* DAPP-PG 672 [19]. As for the genomic DNA G+C content of the OPT-41 strain, it is within the range of values characteristic of the *Erwinia* genus and is closest to that of *E. aphidicola* X 001^T^ (Table 3).

Taking into account all the obtained taxonomic data, strain OPT-41 was assigned to a new species of the genus *Erwinia*, for which the name *E. plantamica* sp. nov. was proposed.

#### 3.1.6. Description of *E. plantamica* sp. nov.

The etymology of the species name “*plantamica*” (plan.ta’.mi.ca) is as follows: L. fem. n. *planta*, plant; L. fem. n. *amica*, friend; L. nom. fem. n.—*plantamica*, friend of plant.

The colony morphology on R2A at 29 °C is circular, smooth, convex, yellowish and translucent, with a diameter of about 4 mm. The pH optimum is 6.5–7.0. The temperature range for growth is 5–44 °C and the optimum temperature is 29 °C. Cells are rod-shaped, Gram-negative, non-endospore-forming, and facultative aerobic (0.9–1.3 × 1.5–3.1 µm). They are catalase-positive and oxidase- and urease-negative. Nitrate is reduced to nitrite. Arginine hydrolase, lysine decarboxylase, lipase, lecithinase, and indole are not produced. This species hydrolyzes esculin, gelatin, and starch. Acid produced from the fermentation was observed in the OF test with glucose, rhamnose, mannose, lactose, maltose, sucrose, cellobiose, arabinose, melibiose, inositol, and sorbitol. Gas formation was observed with maltose, mannose, melibiose, lactose, inositol, and sorbitol. This species produced β-galactosidase, assimilated L-arabinose, D-glucose, D-maltose, D-mannose, D-mannitol, N-acetylglucosamine, potassium gluconate, malic acid, and sodium citrate but did not assimilate capric, adipic acid and phenylacetic acids (according to the API^®^ 20NE tests).

The type strain, OPT-41 (=IBPPM 712=VKM B-3873D=CCTCC AB 2024361), was isolated from seedlings of *Triticum aestivum* L. (var. Simbircite), Kazan, Russia. The genomic DNA G+C content is 55.67%. The GenBank accession for the OPT-41 16S rRNA gene sequence is PQ039748. The GenBank accession for the OPT-41 whole-genome shotgun project is JBGCUC000000000 (BioProject accession, PRJNA1137566; BioSample accession, SAMN42622146; and assembly accession, GCA_043420595.1).

### 3.2. OPT-41 Phytopathogenicity

#### 3.2.1. Extracellular Enzymatic Activities

Most (if not all) phytopathogenic bacteria damage host plant cell walls by producing extracellular plant cell wall-degrading enzymes (PCWDEs) that depolymerize cellulose, pectins, and proteins. Therefore, we analyzed whether the activities of PCWDEs (pectate lyase, polygalacturonase, protease, and cellulase) in the assayed strain *E. plantamica* OPT-41 were comparable to those of the well-known virulent strain *P. atroseptcium* SCRI1043 (formerly *Erwinia carotovora* ssp. *atroseptica* SCRI1043), which causes blackleg and soft rot diseases in plants [53,54].

**Figure 4:**
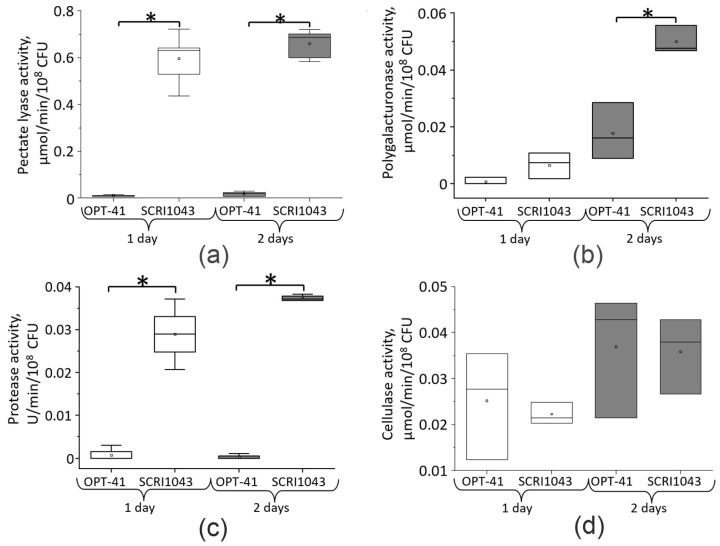
The extracellular pectate lyase (**a**), polygalacturonase (**b**), protease (**c**), and cellulase (**d**) activities measured in the cultural supernatants of *Erwinia plantamica* OPT-41 and *Pectobacterium atroseptcium* SCRI1043 after one day (white box charts) and two days (gray box charts) of cultivation in minimal medium supplemented with wheat extract. Asterisks (*) show the significance of the difference (two-tailed Mann–Whitney test, *p* < 0.05) between variants designated by brackets.

The extracellular pectate lyase activity in *E. plantamica* was 67-fold and 39-fold lower than that in *P. atrosepticum* after one and two days of cultivation, respectively (Figure 4a). The extracellular polygalacturonase activity did not differ significantly in *E. plantamica* and *P. atrosepticum* after one day of cultivation, whereas by the second day, it was 2.9-fold higher in the *P. atrosepticum* cultures (Figure 4b). The extracellular protease activity in *E. plantamica* was 10-fold and 36-fold lower than that in *P. atrosepticum* after one and two days of cultivation, respectively (Figure 4c). No significant differences in extracellular cellulase activity were observed between *E. plantamica* and *P. atrosepticum* on either the first or second day of cultivation (Figure 4d).

Thus, the low levels of extracellular pectate lyase, polygalacturonase, and protease activities in *E. plantamica* compared to the virulent *P. atrosepticum*—where these enzymes are major virulence factors [55,56,57]—indicate that the assayed strain *E. plantamica* OPT-41 is unlikely to possess significant potential for degrading the polymers of the host plant cell wall (pectins and proteins). The absence of differences in the extracellular cellulase activities between *E. plantamica* and *P. atropinism* can likely be attributed to the fact that the latter does not belong to cellulolytic bacteria, and its cellulase activity is rather low despite its high virulence largely conferred by pectolytic and proteolytic enzymes.

On the other hand, the extracellular hydrolases discussed in this study play a multifaceted role in plant-associated bacteria. For example, pectate lyase (PelA) and cellulase (EGY) of the phytopathogenic *Dickeya chrysanthemi* (also known as *Erwinia chrysanthemi*) may be involved in the initial dialog between the bacterium and the plant before the action of other plant tissue-macerating enzymes [58]. Hydrolases secreted by PGPRs may suppress plant pathogens (pectate lyase, polygalacturonase, protease, and cellulase) [59,60] and may decompose organic matter and ensure the circulation of soil nutrients in the rhizosphere (protease and cellulase) [61]. Hydrolase-producing PGPRs have shown their effectiveness as biofertilizers and/or biocontrol agents for promoting growth and health in plants [59,60,61].

#### 3.2.2. Effect of Bacterial Inoculation on Wheat and Barley Seed Germination

**Figure 5:**
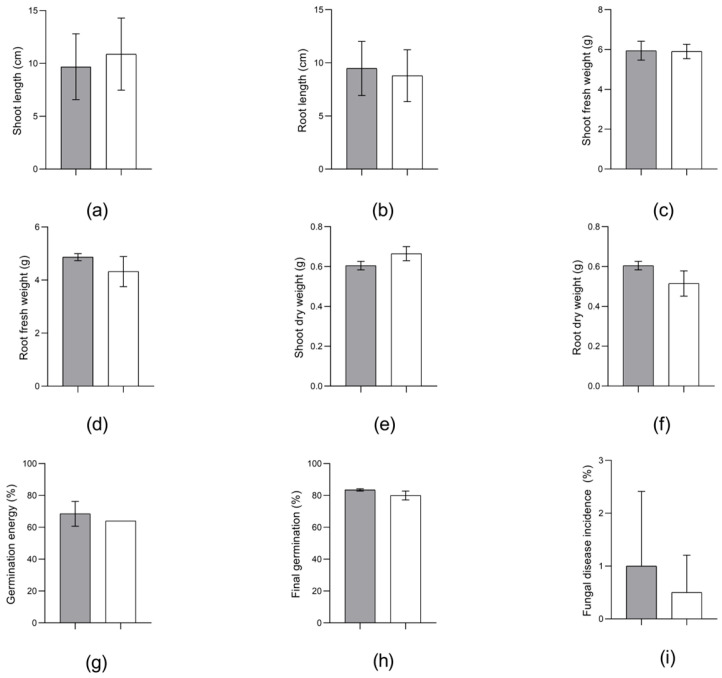
Morphological and physiological parameters of *Triticum aestivum* L. non-inoculated (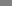) and inoculated (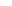) with *Erwinia plantamica* strain OPT-41: (**a**) shoot length, (**b**) root length, (**c**) shoot fresh weight, (**d**) root fresh weight, (**e**) shoot dry weight, (**f**) root dry weight, (**g**) germination energy, (**h**) final germination, and (**i**) fungal disease incidence. The data in the figure are the mean ± SD for shoot and root length and the median ± SD for all others.

**Figure 6:**
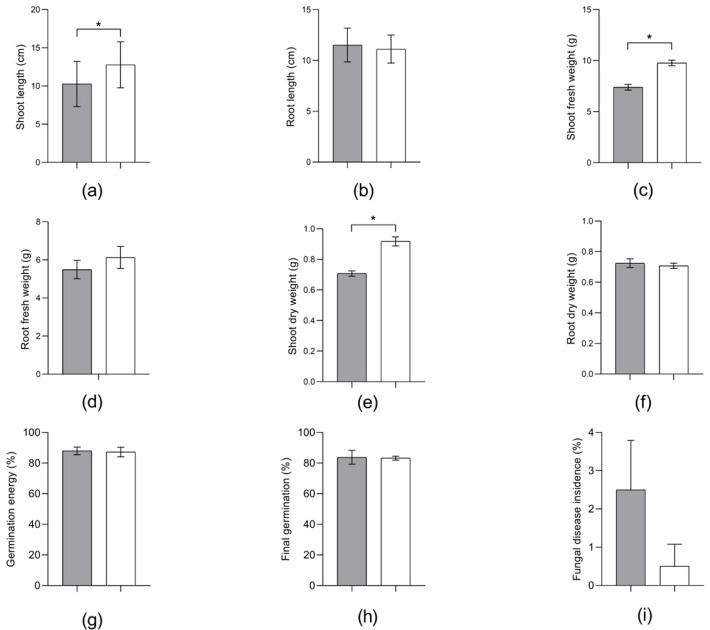
Morphological and physiological parameters of *Hordeum vulgare* L. non-inoculated (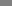) and inoculated (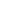) with *Erwinia plantamica* strain OPT-41: (**a**) shoot length, (**b**) root length, (**c**) shoot fresh weight, (**d**) root fresh weight, (**e**) shoot dry weight, (**f**) root dry weight, (**g**) germination energy, (**h**) final germination, and (**i**) fungal disease incidence. The data in the figure are the mean ± SD for shoot and root length and the median ± SD for all others. Asterisks (*) show the significance of the difference between variants designated by brackets (unpaired *t* test, *p* < 0.05, for shoot and root length; two-tailed Mann–Whitney test, *p* < 0.05, for all others).

The traditional approach for assessing the phytopathogenicity of bacteria, particularly belonging to the Erwinia genus [18], involves the in planta inoculation method. The results of the strain OPT-41 testing in relation to spring wheat are presented in Figure 5 and Figure S2, and in relation to spring barley, in Figure 6 and Figure S3.

Statistically significant differences were not observed between the wheat seed treatment with *E. plantamica* strain OPT-41 and the control, and no symptoms of a pathological process (e.g., necrosis, chlorosis, shoot development abnormalities) were detected (Figure 5 and Figure S2).

The treatment of barley seeds with *E. plantamica* strain OPT-41 resulted in a statistically significant increase in the shoot length (Figure 6a) and the shoot fresh (Figure 6c) and dry (Figure 6e) weight by 24, 32 and 30%, respectively. Symptoms of the pathological process were not found in barley (see Figure S3). Previously, positive effects of inoculation with *Erwinia* species were reported for tomato [21], wheat [22], and ginseng [23]. In the case of tomato, plant inoculation with *Erwinia* increased the shoot and root length by 27 and 42%, fresh weight by 50 and 47%, and dry weight by 33 and 37%, respectively [21]. Inoculation with *Erwinia* spp. was also reported to increase the wheat root length by 64% [22] and ginseng shoot length by 15% [23].

The experimental findings indicated a decrease in the incidence of fungal disease in barley seeds treated with *E. plantamica* strain OPT-41 in comparison with the control group. Antifungal activity has been shown in particular for *Pantoea agglomerans*, a close relative of *Erwinia* bacteria, which is known as *E. herbicola* [62,63].

## 4. Conclusions

Based on the results of genome-based taxonomy and traditional polyphasic taxonomy, strain OPT-41 isolated from spring wheat seedlings is a member of the novel *Erwinia* species, for which the name *E. plantamica* sp. nov was proposed. *E. plantamica* OPT-41 was found to have a low potential for degradation of host plant pectins and proteins and to be *Triticum aestivum*- and *Hordeum vulgare*-friendly, leading to the conclusion about its non-phytopathogenic effects on these plant species. In the case of *Hordeum vulgare*, the plant growth-promoting and antifungal effects of this microorganism were noted, indicating the possibility of its use as a biofertilizer and/or biological control agent.

## Figures and Tables

**Table 1:** Pairwise comparisons of the OPT-41 genome against closely related type strain genomes.

Type Strain (Assembly Accession)	dDDH (%) *						Ortho ANIu (%)	G+C Content Difference (%)
	*d_0_*	CI *d_0_*	*d_4_*	CI *d_4_*	*d_6_*	CI *d_6_*		
*Erwinia phyllosphaerae* CMYE1 (GCF_019132875)	44.4	[41.1–47.9]	27.2	[24.8–29.7]	39.0	[36.1–42.1]	83.44	1.91
*Erwinia pyri* DE2 (GCF_030758455)	28.8	[25.5–32.5]	23.2	[20.9–25.7]	26.5	[23.6–29.6]	79.73	1.40
*Erwinia aphidicola* JCM 21238 (GCA_014773485)	23.9	[20.6–27.6]	22.3	[20.1–24.8]	22.6	[19.8–25.7]	78.71	1.00
*Erwinia psidii* IBSBF 435 (GCA_003846135)	20.7	[17.5–24.3]	22.1	[19.9–24.6]	20.0	[17.2–23.0]	77.92	4.19
*Erwinia tasmaniensis* Et1/99 (GCA_000026185)	20.4	[17.2–24.0]	21.8	[19.5–24.2]	19.8	[17.0–22.8]	77.63	2.28
*Mixta gaviniae* DSM 22758 (GCA_002953195)	23.7	[20.4–27.4]	21.8	[19.5–24.2]	22.3	[19.5–25.4]	78.05	2.37
*Candidatus* Erwinia haradaeae EpK1/15 (GCA_002952315)	20.4	[17.2–24.0]	21.8	[19.6–24.3]	19.7	[17.0–22.8]	77.80	2.29
*Mixta calida* LMG 25383 (GCA_002095355)	23.0	[19.7–26.6]	21.7	[19.5–24.2]	21.8	[19.0–24.8]	78.23	1.31
*Erwinia amylovora* CFBP 1232 (GCA_000367625)	19.6	[16.5–23.2]	21.6	[19.4–24.1]	19.1	[16.4–22.1]	77.45	2.10
*Erwinia persicina* NBRC 102418 (GCA_001571305)	21.5	[18.3–25.1]	21.5	[19.2–23.9]	20.6	[17.8–23.6]	77.61	0.26
*Candidatus* Pantoea alvi PSNIH6 (GCA_002920175)	21.2	[18.0–24.8]	21.4	[19.1–23.8]	20.3	[17.6–23.4]	77.32	2.25
*Candidatus* Pantoea bathycoeliae BD_Bin1 (GCA_031332935)	18.5	[15.3–22.0]	21.3	[19.0–23.7]	18.1	[15.4–21.1]	76.91	0.74
*Mixta tenebrionis* BIT-26 (GCA_006517625)	22.3	[19.1–26.0]	21.3	[19.1–23.8]	21.2	[18.4–24.2]	77.66	0.39
*Pantoea vagans* LMG 24199 (GCA_004792415)	19.4	[16.3–23.0]	21.1	[18.8–23.5]	18.9	[16.2–21.9]	76.48	0.33
*Pantoea agglomerans* NBRC 102470 (GCA_001598475)	19.6	[16.4–23.2]	20.7	[18.5–23.1]	19.0	[16.3–22.0]	76.35	0.55
*Pantoea hericii* JZB 2120024 (GCA_014155795)	18.9	[15.8–22.5]	20.5	[18.3–22.9]	18.4	[15.7–21.4]	76.11	1.06
*Candidatus* Pantoea formicae Acro-805 (GCF_011752625)	16.5	[13.5–20.0]	20.1	[17.9–22.5]	16.4	[13.8–19.3]	74.74	2.76
*Pantoea rwandensis* LMG 26275 (GCA_002095475)	16.8	[13.8–20.3]	19.8	[17.6–22.2]	16.6	[14.0–19.6]	74.87	3.06

* Values of the dDDH are given together with their confidence intervals (CIs) for three GBDP formulas, the purpose of which is described in [34].

**Table 2:** Cellular fatty acid profiles of strain OPT-41 and the closest type strain of the genus *Erwinia*.

Fatty Acid	OPT-41	*E. phyllospherae* CMYE1 ^1^
C_12:0_	nd ^2^	4.2
C_14:0_	23.4	5.4
C_16:0_	53.5	32.0
9 C_16:1_	21.1	nd
C_17:0_	nd	0.7
C_17:0_ cyclo	nd	13.5
C_19:0_ cyclo *ω*8*c*	nd	1.8
Summed feature 2 ^3^	nd	9.0
Summed feature 3 ^3^	nd	19.5
Summed feature 8 ^3^	2.0	10.9

^1^ *E. phyllospherae* CMYE1 data are taken from [22], where they are obtained under the same conditions (the reference strain is cultivated on R2A plates at 28 °C for 2 days) using the same method (gas chromatography according to the protocol of the Sherlock Microbial Identification System [43]); ^2^ not determined; ^3^ summed features are fatty acids that cannot be resolved reliably from another fatty acid using the chromatographic conditions chosen. The midi system groups these fatty acids together as one feature with a single percentage of the total. Summed feature 2, C_14:0_ 3-OH and/or C_16:1_ iso I; Summed feature 3, C_16:1_ ω7c and/or C_16:1_ ω6c; Summed feature 8, 9 C_18:1_ and/or 11 C_18:1_.

**Table 3:** Differential characteristics of OPT-41 strain and closely related type strains of *Erwinia* species.

Characteristic	1	2	3	4	5	6
Cell size, µm	0.9–1.3 × 1.5–3.1	0.65–0.86 × 1.2–1.3	na	0.5–0.6 × 1.6–2.0	na	0.5–1.0 × 1.5–2.0
Growth at						
4 °C	−	+	na	na	−	na
37 °C	+	+	−	+	−	−
42 °C	+	+	−	−	−	−
Production:						
nitrate reductase	+	na	na	+	−	−
indole	−	+	−	−	−	−
acetoin	+	na	−	+	−	+
Hydrolysis:						
arginine	−	+	−	−	na	−
gelatin	+	−	−	−	−	−
starch	+	−	na	na	na	−
Assimilation:						
citrate	+	+	+	+	−	+
Acid produced from the fermentation of						
maltose	+	−	na	+	−	na
D-lactose	+	−	na	−	−	na
cellobiose	+	−	na	+	−	na
sorbitol	+	−	+	−	+/−	−
G+C content (%)	55.67	53.76	54.27	56.67	51.48	53.39

Notes: 1—OPT-41 strain; 2—*E. phyllospherae* CMYE1^T^ [22]; 3—*E. pyri* DE2^T^ [16]; 4—*E. aphidicola* X 001^T^ [24], BacDive ID 4403 (https://bacdive.dsmz.de/strain/4403, accessed on 22 December 2024); 5—*E. psidii* DSM 17597^T^, BacDive ID 4397 (https://bacdive.dsmz.de/strain/4397, 22 December 2024); 6—*E. tasmaniensis* Et1/99^T^ [17], BacDive ID 4400 (https://bacdive.dsmz.de/strain/4400, 22 December 2024). G+C content values are calculated directly from completely sequenced genomes (assembly accessions are in the manuscript text and in Table S1); “+”—positive; “−”—negative; na—not available.

## Data Availability

The whole-genome shotgun sequencing project and the 16S rRNA gene sequence presented in this study are openly available in GenBank under the accession numbers JBGCUC000000000 and PQ039748, respectively.

## Supplementary Materials

The following supporting information can be downloaded at: https://www.mdpi.com/article/10.3390/microorganisms13030474/s1, Figure S1: Results of API^®^20NE test in 48 h for strain OPT-41; Figure S2: Seven-day seedlings of *Triticum aestivum* L.; Figure S3: Seven-day seedlings of *Hordeum vulgare* L.; Table S1: TYGS type strains closely related to strain OPT-41 (accessed on 15 July 2024). References [64,65,66,67,68,69,70,71,72,73] are cited in the Supplementary Materials.
