# Supplementary material for: Erwinia plantamica sp. nov., a Non-Phytopathogenic Bacterium Isolated from the Seedlings of Spring Wheat (Triticum aestivum L.)
Source: Microorganisms. 2025 Feb 20;13(3):474. doi: 10.3390/microorganisms13030474 (PMC11944495; doi:10.3390/microorganisms13030474)
Supplement: Supplementary file 1 [file microorganisms-13-00474-s001.zip › Figures S1-S3.pdf]

Supplementary figures

"Erwinia plantamica **sp. nov., a Non-Phytopathogenic Bacterium Isolated from the Seedlings of Spring Wheat (*Triticum aestivum* L.)" by Egorshina et al.**

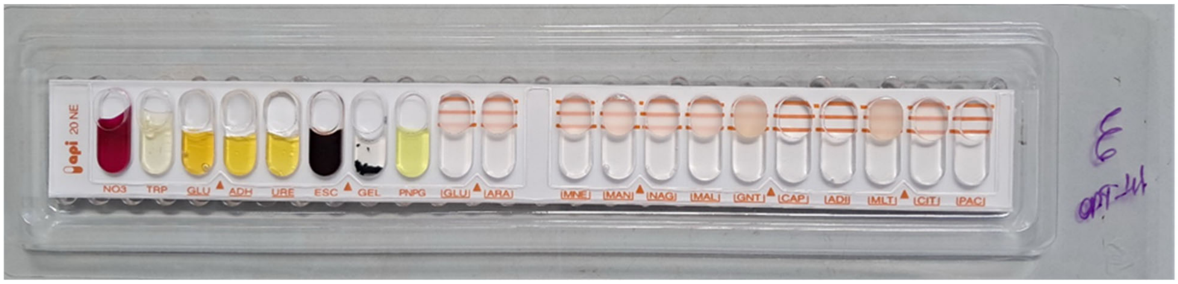

Figure S1. Results of API®20NE test in 48 h for strain OPT-41.

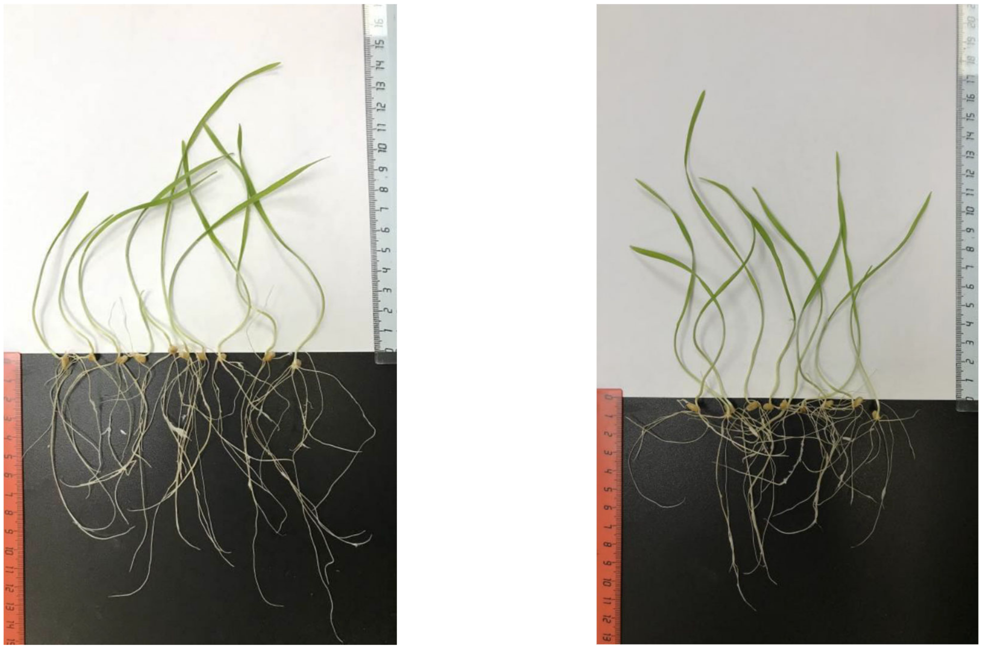

(a) (b)

Figure S2. Seven-day seedlings of *Triticum aestivum* L.: (**a**) non-inoculated and (**b**) inoculated with *Erwinia plantamica* strain OPT-41.

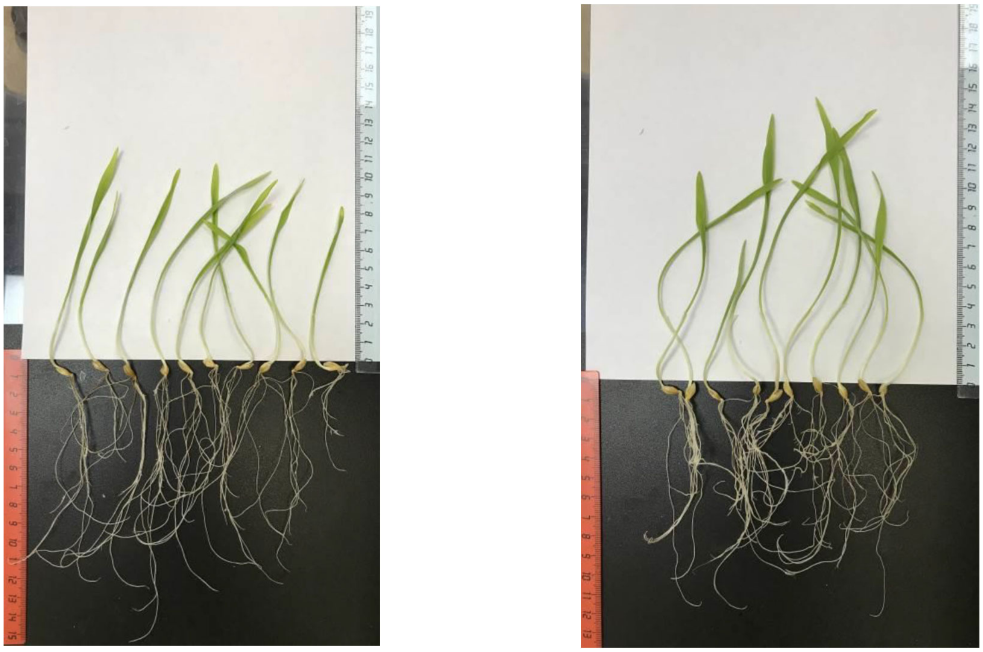

(a) (b)

Figure S3. Seven-day seedlings of *Hordeum vulgare* L.: (**a**) non-inoculated and (**b**) inoculated with *Erwinia plantamica* strain OPT-41.
