# Supplementary material for: Erwinia plantamica sp. nov., a Non-Phytopathogenic Bacterium Isolated from the Seedlings of Spring Wheat (Triticum aestivum L.)
Source: Microorganisms. 2025 Feb 20;13(3):474. doi: 10.3390/microorganisms13030474 (PMC11944495; doi:10.3390/microorganisms13030474)
Supplement: Supplementary file 1 [file microorganisms-13-00474-s001.zip › Table S1.pdf]

Supplementary table

"*Erwinia plantamica* sp. nov., a Non-Phytopathogenic Bacterium Isolated from the Seedlings of Spring Wheat (*Triticum aestivum* L.)" by Egorshina et al.

Table S1. TYGS type-strains closely related to strain OPT-41 (accessed on 15 July 2024)

Strain	TYGS ID	Authority	Other deposits	Synonyms	Base pairs	G+C, %	No. proteins	Goldstamp	Bioproject ID	Biosample ID	Assembly ID
Erwinia phyllosphaerae CMYE1~~T~~	85300	Pan et al. 2022	JCM 34792; GDMCC 1.2674	*Erwinia phyllosphaerae*	4,726,038	53.76	4281	na	PRJNA224116	SAMN19908003	GCF_019132875
Erwinia pyri DE2	25571	He et al. 2024	na	*Erwinia pyri*	4,758,430	54.27	4424	na	PRJNA224116	SAMN36511384	GCF_030758455
Erwinia aphidicola JCM 21238	132009	Harada et al. 1997	LMG 24877; CIP 106296; DSM 19347; NBRC 102417; IAM 14479; X 001	*Erwinia aphidicola*	5,112,699	56.67	4578	na	PRJNA663353	SAMN16130814	GCA_014773485
Erwinia psidii IBSBF 435	19470	Rodrigues Neto et al. 1987	CFBP 3627; LMG 7039; CIP 105200; NCPPB 3555; ATCC 49406; DSM 17597; PDDCC 8426	*Erwinia psidii*	4,497,306	51.48	3954	na	PRJNA498492	SAMN10305463	GCA_003846135
Erwinia tasmaniensis Et1/99	574	Geider et al. 2006	NCPPB 4357; DSM 17950	*Erwinia tasmaniensis*	4,067,864	53.39	3622	Gp0001395	PRJEA20585	SAMEA2272215	GCA_000026185
Mixta gaviniae DSM 22758	18942	(Popp et al. 2010) Palmer et al. 2018	A18/07; LMG 25382	*Mixta gaviniae; Pantoea gaviniae*	4,527,605	58.04	3935	Gp0307784	PRJNA430362	SAMN08369925	GCA_002953195
Candidatus Erwinia haradaeae EpK1/15	122765	Manzano-Marín et al. 2020	na	*Candidatus* Erwinia haradaeae	4,075,681	53.38	3567	na	PRJNA411857	SAMN07689503	GCA_002952315
Mixta calida LMG 25383	22176	(Popp et al. 2010) Palmer et al. 2018	1400/07; DSM 22759	*Mixta calida; Pantoea calida*	4,239,526	56.98	3543	Gp0031805	PRJNA252985	SAMN05757634	GCA_002095355
Erwinia amylovora CFBP 1232	3005	(Burrill 1882) Winslow et al. 1920 emend. Hauben et al. 1998	LMG 2024; CIP 82.82; NCPPB 683; ICMP 1540; ATCC 15580; DSM 30165; IFO 12687; NBRC 12687; NCAIM B.01108	*Erwinia amylovora; Micrococcus amylovorus*	3,795,524	53.57	3762	Gp0042360	PRJEB604	SAMEA2271944	GCA_000367625
Erwinia persicina NBRC 102418	4080	Hao et al. 1990	AJ 2716; CFBP 3622; LMG 11254; CIP 105199; NCPPB 3774; ICMP 12532; ATCC 35998; DSM 19328; JCM 3704; CDC 9108-82; HK 204; IAM 12843	*Erwinia persicina*	4,905,774	55.41	4539	Gp0023679	PRJDB368	SAMD00046721	GCA_001571305

Candidatus Pantoea alvi PSNIH6	133355	Crosby et al. 2023	PSNIH6Ts	*Candidatus* Pantoea alvi	5,344,485	57.92	4995	na	PRJNA430813	SAMN04287067	GCA_002920175
Candidatus Pantoea bathycoeliae BD_Bin1	97460	Fourie et al. 2023	na	*Candidatus* Pantoea bathycoeliae	3,145,092	54.93	4354	na	PRJNA987703	SAMN36078333	GCA_031332935
Mixta tenebrionis BIT-26	16510	Xia et al. 2020	CGMCC 1.17041; KCTC 72449	*Mixta tenebrionis*	4,648,399	56.06	4164	na	PRJNA550228	SAMN12116280	GCA_006517625
Pantoea vagans LMG 24199	16257	Brady et al. 2009	BCC 105; DSM 23078; BD 765; R-21566	*Pantoea vagans*	4,790,329	55.34	4283	Gp0441667	PRJNA505269	SAMN10414043	GCA_004792415
Pantoea agglomerans NBRC 102470	4084	(Beijerinck 1888) Gavini et al. 1989	CFBP 3845; LMG 1286; CIP 57.51; ICMP 12534; ATCC 27155; CCUG 539; DSM 3493; JCM 1236; NCTC 9381; CDC 1461-67; ICPB 3435	*Bacillus agglomerans; Enterobacter agglomerans; Pantoea agglomerans*	4,652,040	55.12	4246	Gp0023598	PRJDB388	SAMD00046726	GCA_001598475
Pantoea hericii JZB 2120024T	25008	Rong et al. 2016	LMG 28847; CGMCC 1.15224	*Pantoea hericii*	4,006,248	54.61	3705	na	PRJNA515154	SAMN10743378	GCA_014155795
Candidatus Pantoea formicae Acro-805	133352	Crosby et al. 2023	Acro-805Ts	*Candidatus* Pantoea formicae	5,887,503	52.91	5536	na	PRJNA224116	SAMN12703576	GCF_011752625
Pantoea rwandensis LMG 26275	22112	Brady et al. 2012	BCC 571; BCC 571 (Bacterial Culture Collection Forestry and Agricultural Biotechnology Institute South Africa); DSM 26585; BD 944	*Pantoea rwandensis*	5,775,402	52.61	4986	Gp0044318	PRJNA252996	SAMN05907793	GCA_002095475

Notes: na – not available

References:

1. Pan, M.K.; Feng, G.D.; Yao, Q.; Li, J.; Liu, C.; Zhu, H. *Erwinia phyllosphaerae* sp. nov., a novel bacterium isolated from phyllosphere of pomelo (*Citrus maxima*). *Int. J. Syst. Evol. Microbiol.* **2022**, *72*, 5316. https://doi.org/10.1099/ijsem.0.005316
2. He, L.; Huang, R.; Chen, H.; Zhao, L.; Zhang, Z. Discovery and characterization of a novel pathogen *Erwinia pyri* sp. nov. associated with pear dieback: taxonomic insights and genomic analysis. *Front. Microbiol.* **2024**, *15*, 1365685. https://doi.org/10.3389/fmicb.2024.1365685
3. Harada, H.; Oyaizu, H.; Kosako, Y.; Ishikawa, H. *Erwinia aphidicola*, a new species isolated from pea aphid, *Acyrthosiphon pisum*. *J. Gen. Appl. Microbiol.* **1997**, *43*, 349–354. https://doi.org/10.2323/jgam.43.349
4. Rodrigues Neto, J.; Robbs, C.F.; Yamashiro, T. A bacterial disease of guava (*Psidium guajava*) caused by *Erwinia psidii* sp. nov. *Fitopatol. Bras.* **1987**, *12*, 345–350.
5. Geider, K.; Auling, G.; Du, Z.; Jakovljevic, V.; Jock, S.; Volksch, B. *Erwinia tasmaniensis* sp. nov., a non-phytopathogenic bacterium from apple and pear trees. *Int. J. Syst. Evol. Microbiol.* **2006**, *56*, 2937–2943. https://doi.org/10.1099/ijs.0.64032-0

6. Popp, A.; Cleenwerck, I.; Iversen, C.; De Vos, P.; Stephan, R. *Pantoea gaviniae* sp. nov. and *Pantoea calida* sp. nov., isolated from infant formula and an infant formula production environment. *Int J Syst Evol Microbiol.* **2010**, *60*, 2786–2792. https://doi.org/10.1099/ijs.0.019430-0
7. Palmer, M; Steenkamp, E.T.; Coetzee, M.P.A.; Avontuur, J.R.; Chan, W.Y.; van Zyl, E.; Blom, J.; Venter, S.N. *Mixta* gen. nov., a new genus in the *Erwiniaceae*. *Int. J. Syst. Evol. Microbiol.* **2018**, *68*, 1396–1407. https://doi.org/10.1099/ijsem.0.002540
8. Manzano-Marin, A.; Coeur d'acier, A.; Clamens, A.L.; Orvain, C.; Cruaud, C.; Barbe, V.; Jousselin, E. Serial horizontal transfer of vitamin-biosynthetic genes enables the establishment of new nutritional symbionts in aphids' di-symbiotic systems. *ISME J*. **2020**, *14*, 259–273. https://doi.org/10.1038/s41396-019-0533-6
9. Winslow, C.E.; Broadhurst, J.; Buchanan, R.E.; Krumwiede, C.; Rogers, L.A.; Smith, G.H. The Families and Genera of the Bacteria: Final Report of the Committee of the Society of American Bacteriologists on Characterization and Classification of Bacterial Types. *J. Bacteriol.* **1920**, *5*, 191–229. https://doi.org/10.1128/jb.5.3.191-229.1920.
10. Hauben, L.; Moore, E.R.; Vauterin, L.; Steenackers, M.; Mergaert, J.; Verdonck, L.; Swings, J. Phylogenetic position of phytopathogens within the *Enterobacteriaceae*. *Syst. Appl. Microbiol.* **1998**, *21*, 384–397. https://doi.org/10.1016/S0723-2020(98)80048-9
11. Hao, M.V.; Brenner, D.J.; Steigerwalt, A.G.; Kosako, Y.; Komagata, K. *Erwinia persicinus*, a new species isolated from plants. *Int. J. Syst. Bacteriol.* **1990**, *40*, 379–383. https://doi.org/10.1099/00207713-40-4-379
12. Crosby, K.C.; Rojas, M.; Sharma, P.; Johnson, M.A.; Mazloom, R.; Kvitko, B.H.; Smits, T.H.M.; Venter, S.N.; Coutinho, T.A.; Heath, L.S.; Palmer, M.; Vinatzer, B.A. Genomic delineation and description of species and within-species lineages in the genus *Pantoea*. *Front. Microbiol*. **2023**, *14*, 1254999. https://doi.org/10.3389/fmicb.2023.1254999
13. Fourie, A.; Venter, S.N.; Slippers, B.; Fourie, G. *Pantoea bathycoeliae* sp. nov and *Sodalis* sp. are core gut microbiome symbionts of the two-spotted stink bug. *Front. Microbiol*. **2023**, *14*, 1284397. https://doi.org/10.3389/fmicb.2023.1284397
14. Xia, M.; Wang, J.; Huo, Y.X.; Yang, Y. *Mixta tenebrionis* sp. nov., isolated from the gut of the plastic-eating mealworm *Tenebrio molitor* L. *Int. J. Syst. Evol. Microbiol*. **2020**, *70*, 790–796. https://doi.org/10.1099/ijsem.0.003826
15. Brady, C.L.; Venter, S.N.; Cleenwerck, I.; Engelbeen, K.; Vancanneyt, M.; Swings, J.; Coutinho, T.A. *Pantoea vagans* sp. nov., *Pantoea eucalypti* sp. nov., *Pantoea deleyi* sp. nov. and *Pantoea anthophila* sp. nov. *Int. J. Syst. Evol. Microbiol*. **2009**, *59*, 2339–2345. https://doi.org/10.1099/ijs.0.009241-0
16. Gavini, F.; Mergaert, J.; Beji, A.; Mielcarek, C.; Izard, D.; Kersters, K.; De Ley, J. Transfer of *Enterobacter agglomerans* (Beijerinck 1888) Ewing and Fife 1972 to *Pantoea* gen. nov. as *Pantoea agglomerans* comb. nov. and description of *Pantoea dispersa* sp. nov. *Int. J. Syst. Bacteriol*. **1989**, *39*, 337–345. https://doi.org/10.1099/00207713-39-3-337
17. Rong, C.; Ma, Y.; Wang, S.; Liu, Y.; Chen, S.; Huang, B.; Wang, J.; Xu, F. *Pantoea hericii* sp. nov., Isolated from the Fruiting Bodies of *Hericium erinaceus*. *Curr. Microbiol*. **2016**, *72*, 738–743. https://doi.org/10.1007/s00284-016-1011-2
18. Brady, C.L.; Cleenwerck, I.; van der Westhuizen, L.; Venter, S.N.; Coutinho, T.A.; De Vos, P. *Pantoea rodasii* sp. nov., *Pantoea rwandensis* sp. nov. and *Pantoea wallisii* sp. nov., isolated from Eucalyptus. *Int. J. Syst. Evol. Microbiol*. **2012**, *62*, 1457–1464. https://doi.org/10.1099/ijs.0.032615-0
